# The ‘Dream Team’ for sexual, reproductive, maternal, newborn and adolescent health: an adjusted service target model to estimate the ideal mix of health care professionals to cover population need

**DOI:** 10.1186/s12960-017-0221-4

**Published:** 2017-07-04

**Authors:** Petra ten Hoope-Bender, Andrea Nove, Laura Sochas, Zoë Matthews, Caroline S. E. Homer, Francisco Pozo-Martin

**Affiliations:** 1 Technical Adviser Sexual and Reproductive Health and Rights, United Nations Population Fund, Geneva, Switzerland; 2 Instituto de Cooperación Social Integrare, Calle Balmes, 30, 3° - 1, 08007 Barcelona, Spain; 3 Novametrics Ltd, Derby, United Kingdom; 4 London School of Economics, London, United Kingdom; 5 Department of Social Statistics and Demography, University of Southampton, Southampton, United Kingdom; 6 Centre for Midwifery, Child and Family Health, University of Technology Sydney, Ultimo, NSW Australia; 7 Department of Global Health and Development, Faculty of Public Health, London School of Hygiene and Tropical Medicine, London, United Kingdom

**Keywords:** Health workforce planning, Human resources for health, Midwifery, Sexual, reproductive, maternal, newborn and adolescent health, Universal health coverage, Sustainable development goals

## Abstract

**Background:**

A competent, enabled and efficiently deployed health workforce is crucial to the achievement of the health-related sustainable development goals (SDGs). Methods for workforce planning have tended to focus on ‘one size fits all’ benchmarks, but because populations vary in terms of their demography (e.g. fertility rates) and epidemiology (e.g. HIV prevalence), the level of need for sexual, reproductive, maternal, newborn and adolescent health (SRMNAH) workers also varies, as does the ideal composition of the workforce. In this paper, we aim to provide proof of concept for a new method of workforce planning which takes into account these variations, and allocates tasks to SRMNAH workers according to their competencies, so countries can assess not only the needed size of the SRMNAH workforce, but also its ideal composition (the ‘Dream Team’).

**Methods:**

An adjusted service target model was developed, to estimate (i) the amount of health worker time needed to deliver essential SRMNAH care, and (ii) how many workers from different cadres would be required to meet this need if tasks were allocated according to competencies. The model was applied to six low- and middle-income countries, which varied in terms of current levels of need for health workers, geographical location and stage of economic development: Azerbaijan, Malawi, Myanmar, Peru, Uzbekistan and Zambia.

**Results:**

Countries with high rates of fertility and/or HIV need more SRMNAH workers (e.g. Malawi and Zambia each need 44 per 10,000 women of reproductive age, compared with 20–27 in the other four countries). All six countries need between 1.7 and 1.9 midwives per 175 births, i.e. more than the established 1 per 175 births benchmark.

**Conclusions:**

There is a need to move beyond universal benchmarks for SRMNAH workforce planning, by taking into account demography and epidemiology. The number and range of workers needed varies according to context. Allocation of tasks according to health worker competencies represents an efficient way to allocate resources and maximise quality of care, and therefore will be useful for countries working towards SDG targets. Midwives/nurse-midwives who are educated according to established global standards can meet 90% or more of the need, if they are part of a wider team operating within an enabled environment.

## Background

The inclusion of the health workforce as a key strategy to achieve Sustainable Development Goal (SDG) 3 on health [1], and the global strategy on human resources for health (HRH) [2] are signs of a greater emphasis on the health workforce as an engine for human development [3]. The 2015 report of the independent Expert Review Group [4] called for the delivery of ‘an expanded and skilled health workforce, especially in Sub-Saharan Africa, which serves women and children with measurable impact’ and its 2014 report clearly identified the lack of health workers (especially midwives) as a major neglected global and national priority [5]. The 2014 State of the World’s Midwifery (SoWMy 2014) report [6] provided new data on the midwifery workforce to inform policy and planning with SRMNAH workforce projections for 73 countries based on full-time equivalent (FTE) staff rather than headcounts. The 2014 Lancet Series on Midwifery established a framework for quality maternal and newborn care (QMNC), the package of care required and the values and philosophy embodied in this care are key to achieving the post-2015 vision to end preventable maternal and newborn deaths and improve health and wellbeing [7].

Acting on such recommendations requires evidence about the size, distribution and, crucially, skill mix of human resources for health (HRH). Past efforts to estimate HRH needs have tended to use workforce-to-population ratios, typically a number of doctors, nurses and midwives per 10,000 population [8, 9]. Such ratios have many advantages: they are simple to calculate, are easy to communicate and are derived from observed data. On the other hand, they assume that needs are similar across countries or sub-national areas, they are usually based on headcounts of health workers as opposed to FTEs, and most do not specify either the type and skills of health workers that are required [10], nor options for configuring teams of health workers with the appropriate skill mix. While potentially useful, they ‘are not a substitute for specific country assessments of sufficiency’ in terms of the size and skill mix of the health workforce [8]. SDG 3 calls for an adequate distribution of HRH, which can be interpreted geographically, but also across cadres and full-time/part-time workers. Given new developments in the availability of health workforce data, the disaggregation of demographic indicators at the 100-m^2^ level worldwide [11], and a consensus on the essential interventions needed [12], the time is right for a new approach to workforce planning that better enables countries to achieve equitable and effective coverage of SRMNAH services.

This paper presents a method for workforce planning that identifies an ideal, country-specific team of health care professionals to take care of women of reproductive age and newborns, based on their competencies. We call this team the *Dream Team* 1 because the method considers the best fit of provider competencies at each level of the health care system.

The method takes as its basis the SoWMy 2014 report’s workforce modelling exercise [6], which allocated the time taken to deliver essential SRMNAH interventions to the country’s existing cadres of SRMNAH worker according to the principle of economic efficiency. In this paper, the SoWMy 2014 model has been modified to estimate how many and which types of HRH would be needed to deliver universal coverage of essential SRMNAH interventions if tasks are allocated among different cadres according to the best fit between the nature of the task and each cadre’s competencies. The model also incorporates the QMNC-effective practices identified in the Lancet series on Midwifery [7]. This new approach considers need for SRMNAH workers across the whole continuum of care, according to the specific demographic and epidemiological profile of the country or region in question, in contrast to the ‘175 births per midwife’ threshold put forward in the 2005 World Health Report [13].

For each of six selected countries, the results presented in this paper answer the following questions:

1. How many health care professionals are necessary to meet the population need for SRMNAH services?
2. What mix of different cadres of health care professional would ideally enable the country to meet the need for SRMNAH services, if tasks were to be allocated according to competencies?
3. How does the ideal configuration of the SRMNAH team vary at different stages of the continuum of care (pre-pregnancy, pregnancy, labour/birth and postnatal) and at different levels of the health system (primary, secondary, tertiary)?

The results for the six countries use 2012 data in order to test the method, so the results are applicable to 2012 rather than to the present day. More precise estimates would require up-to-date and accurate data on the country’s health workforce, demography and epidemiology.

This paper provides an important addition to the literature because it proposes a new approach to workforce planning that overcomes the criticisms of workforce-to-population ratios and is feasible to implement for countries with limited HRH data. Although the method and analysis presented in this paper relate specifically to the SRMNAH workforce, the same principles could be applied to other sections of the health workforce. The method could therefore help countries achieve SDG 3 and the other health-related targets, as well as implement the new global HRH strategy [2]. It could also be adapted to provide sub-national estimates, to help address the SDG equity agenda.

## Methods

A deterministic mathematical model was developed to estimate the number and relative contribution of the different SRMNAH cadres necessary to provide universal coverage of key SRMNAH interventions in a given country in a particular year. The approach involves estimating the number of overall annual full-time equivalents (FTEs) required to deliver each intervention and then assigning the task of providing each intervention to a cadre with the required competencies (see Annex A2 for details of tasks), resulting in quality care plus economic efficiency in terms of SRMNAH workforce requirements.

Before commencing the modelling, we defined the key interventions representing need for SRMNAH care from two evidence-based frameworks: (1) the list of essential interventions proposed by the Partnership for Maternal, Newborn and Child Health (PMNCH) [12], supplemented by (2) practices identified as effective in the 2014 Lancet Series on Midwifery [7]. The full list of interventions is shown in Appendix Table 6.

Implementing the approach for a specific country involves three steps, each corresponding to one of the three research questions outlined above:

- Estimate the total number of FTEs required to provide universal coverage of all the key interventions in a given year (in this case, 2012). This step requires:
- Estimating the number of women, girls and babies requiring each intervention in that year using demographic and epidemiological data taken from the SoWMy 2014 dataset, which included 73 of the 75 ‘Countdown to 2015’ countries [14]. Most of the demographic data are country-specific, whereas most of the epidemiological data are regional averages. The reference year for this dataset is 2012. The SoWMy 2014 analysis was based on the PMNCH essential interventions and for these interventions, details of data sources can be found in Annex 4 of the SoWMy 2014 report [6]. For the additional effective practices included for this paper, details of data sources can be found in Appendix Table 8.
- Estimating the contact time (in minutes, shown in Appendix Table 6) required to deliver each intervention to one individual, using time estimates from the OneHealth tool [15] and, where unavailable, estimates based on expert opinion.
- Based on the previous two quantities, estimating the total annual contact time (in hours) required to deliver each intervention to all the individuals who need it.
- Translating the total annual hours required to deliver each intervention into the equivalent number of FTEs, where a FTE worker was assumed to spend 1880 h working per year (i.e. assuming all SRMNAH workers work 40 h per week, take an average of 5 days of sick leave and 20 days of paid annual leave per year according to International Labour Organisation standards [16]); of this time, the worker spends 70% of their available working hours (i.e. 1316 h) providing clinical interventions as opposed to administrative tasks and other duties. Hence, in order to obtain the number of FTEs, one must divide total clinical hours required by 1316.
- Summing the number of FTEs needed to deliver universal coverage of all the interventions.

Table 1 shows an example of stages (a) to (d) for one intervention: external cephalic version (an intervention which attempts to turn the foetus into the optimal ‘head-down’ position before labour commences, if it is not already in this position).

**Table 1:** Illustration of method used to estimate the number of FTEs required to deliver external cephalic version (ECV)

Annual number of births (stage a: demographic data)	100 000
Prevalence of breech presentation at birth (stage a: epidemiological data)	4.3%
Annual number of breech presentations (stage a: demographic and epidemiological data)	4.3% of 100 000 = 4 300
Time required for one ECV (stage b)	107 min^a^
Total annual time required (stage c)	460 100 min = 7 668 h
Total annual FTEs required (stage d)	7 668/1 316 = 5.83 FTEs

NB These data are for illustrative purposes only; they are not taken from a particular country

^a^The time includes preparation (ultrasound to confirm presentation), counselling and consent, foetal monitoring pre- and post-procedure, and follow-up

- Allocate the annual number of FTE workers required to deliver each intervention to a cadre with the relevant competencies. This step involves:
- Defining the health care professional cadres responsible for providing the SRMNAH interventions and assigning them to one of five core categories of health worker as listed in Table 2. The same five categories are used for all countries, regardless of whether or not that cadre exists (or has that name, or has a particular set of competencies) in all countries, because the aim is to describe the ideal configuration of the SRMNAH workforce.

**Table 2:** Cadre categories and ordering from lowest to highest salary

Cadre category	Ordering from lowest to highest paid
Auxiliary midwives and nurse midwives^a^	1 (lowest)
Midwives and nurse-midwives^a^	2
Medical officers^b^	3
Doctors (generalists)	4
Doctors (obstetricians/gynaecologists)	5 (highest)

^a^For the purpose of this paper nurse-midwife relates to the education trajectory of becoming a nurse first and then qualifying as a midwife, allowing for deployment in many parts of the health system. The decision to merge the nurse-midwife and midwife cadres was taken because in some countries there is no distinction between the two, and in other countries they have similar or identical competencies. In countries with a clear distinction between the roles and responsibilities of midwives and nurse-midwives, it may be more appropriate to treat them as two separate cadres

^b^Not all countries have a medical officer cadre, in which case the tasks allocated to medical officers in this analysis would be allocated to a generalist doctor cadre
- Determining which of the SRMNAH interventions each cadre category is competent to perform based on the International Standard Classification of Occupations [17] and Optimize MNH [18]—see Appendix Table 7.
- Using a logical algorithm, allocating sequentially to each cadre category the number of FTEs required to provide universal coverage for each intervention based on whether or not that cadre is competent to deliver that intervention. If two or more cadres are competent to deliver a particular intervention, all of the FTEs required are allocated to the lowest paid of the competent cadres (i.e. the one appearing highest in Table 2) to maximise economic efficiency as well as quality of care.
- Estimate the annual number of FTEs of each cadre category required to deliver the SRMNAH interventions overall, by stage on the SRMNAH continuum of care (pre-pregnancy, pregnancy, labour/birth, postnatal), and by institutional level of care (primary, secondary, tertiary).
  This step requires the aggregation of cadre category-specific FTEs across (a) all interventions and (b) all interventions within each stage of the SRMNAH continuum. In addition, it requires assumptions about the level of care at which each intervention is delivered (see Appendix Table 6). For interventions provided at all three levels (e.g. if more severe cases are referred to higher levels of care), it was assumed that 60% of the time requirement would be delivered at primary level, 30% at secondary level and 10% at tertiary level. (Because the assumption about the proportion of the time required at different levels of health system was not evidence-based, we also modelled a 50-30-20 split and a 70-20-10 split. For Azerbaijan, Uzbekistan, Myanmar and Peru it made very little difference due to relatively low incidence of conditions such as HIV and malaria in these countries, which means that there is relatively little need for SRMNAH services at secondary and tertiary levels. For Malawi and Zambia, a 10 percentage point decrease in the proportion of working time allocated to primary care was associated with a 6 percentage point decrease in the proportion of FTEs needed at primary level. Under all three scenarios, however, it was clear that the majority of the need for SRMNAH workers is at the primary level of care.)

Future projections of workforce need were based on the method as described above, but using projections of the numbers of women of reproductive age and the number of births and pregnancies in each country, for each year up to 2030. Projections of the numbers of women of reproductive age were taken from the United Nations population database 2012 revision (medium fertility assumption). Birth and pregnancy projections were provided by the Geodata Institute at the University of Southampton [11] according to the methodology described in Annex 6 of the SoWMy 2014 report [6]. The future projections assumed that the epidemiological conditions (e.g. HIV prevalence, malaria prevalence) current in 2012 would continue to apply through to 2030.

### Community health workers

Even though many countries deploy community health workers (CHWs) to perform SRMNAH tasks, they were not counted as part of the SRMNAH workforce because there is no standard definition of a CHW nor a standard expectation of which SRMNAH tasks they are competent and authorised to deliver. This limitation should be borne in mind when interpreting the analysis of workforce availability. WHO is currently working towards addressing this issue [19], so in future years it may be feasible to include CHWs in workforce modelling exercises.

We applied the three stages described above to data from 2012 for six countries. The countries were selected in a two-stage process (Table 3). First, the 73 SoWMy 2014 countries were allocated to three groups according to their 2015 Human Development Index (HDI) classification [20]: low HDI (below the 33% HDI percentile), medium (above the 33% HDI percentile and below the 66% HDI percentile) and high HDI (above the 66% HDI percentile). From each of the three groups, we selected the country with the highest level of SRMNAH workforce need (according to SoWMy 2014) and the country with the lowest.

**Table 3:** Country selection

	Lowest need for SRMNAH workers	Highest need for SRMNAH workers
High HDI group	Azerbaijan	Peru
Medium HDI group	Uzbekistan	Zambia
Low HDI group	Myanmar	Malawi

The six selected countries are diverse with respect to their current levels of need for SRMNAH workers, geography and stage of economic development. The countries also represent a range of stages in the Obstetric Transition [21]. This variety allows an assessment of how well the new method applies in different low- and middle-income settings. Table 4 shows some key demographic and epidemiological indicators for the selected countries.

**Table 4:** Demographic and epidemiological indicators for the six selected countries

Country	Stage in obstetric transition^f^	Maternal mortality ratio, 2015^a^	Neonatal mortality rate, 2015^b^	Total fertility rate, 2010–2015^c^	Prevalence of HIV in adults aged 15–49, 2012^d^	Contraceptive method mix^g^: % female sterilisation^e^
Azerbaijan	IV	25	18	2.30	0.1	0.8
Malawi	II	634	22	5.25	10.9	21.0
Myanmar	III	178	26	2.25	0.7	2.2
Peru	III	68	8	2.50	0.4	10.7
Uzbekistan	IV	36	20	2.48	0.2	3.2
Zambia	III	224	21	5.45	12.8	3.9

Sources: ^a^WHO et al [29]; ^b^Healthy Newborn Network [30]; ^c^UN Population Division [31]; ^d^UNAIDS [32]; ^e^Azerbaijan 2006 DHS [33], Malawi 2010 DHS [34], Myanmar MoH [35], Peru 2012 DHS [36], Uzbekistan 2006 MICS [37], Zambia 2013-14 DHS [38]

^f^I: maternal mortality ratio (MMR) > 1000; II: MMR 300–999; III: MMR = 50–299; IV: MMR <50; V: no avoidable deaths

^g^Currently married women

We express the ideal size and configuration of the SRMNAH workforce in terms of the number of health care professionals needed per 10,000 women of reproductive age (15–49 years), because women of reproductive age are the main user group of SRMNAH services. According to SoWMy 2014, the majority of the need for SRMNAH services occurs outside of pregnancy (e.g. family planning services, prevention and management of sexually transmitted infections), hence the use of women of reproductive age as a demographic reference category rather than the number of pregnancies or births. We do, however, calculate the number of midwives needed per 175 births to enable a comparison against this established benchmark.

## Results

**Fig. 1:**
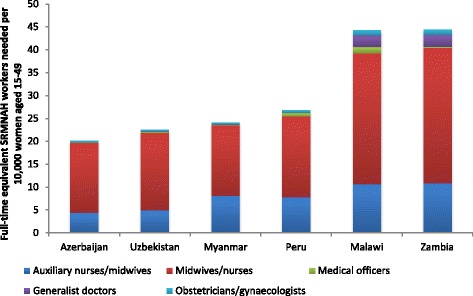
Number of full-time equivalent SRMNAH workers needed per 10,000 women of reproductive age, 2015

The number of FTE SRMNAH workers needed per 10,000 women of reproductive age varied according to the different levels of need in the six countries, as illustrated by Fig. 1. In Malawi and Zambia, the number of health workers needed is much greater (44 FTEs needed per 10,000 women of reproductive age), which reflects the relatively high levels of fertility and HIV prevalence in these countries (see Table 4). The need for FTEs in these two countries is roughly double the number needed per 10,000 women of reproductive age in Azerbaijan, Myanmar and Uzbekistan (20, 24 and 23 respectively) and still considerably higher than the 27 needed in Peru.

These results indicate a higher level of need for SRMNAH workers than was suggested in the SoWMy 2014 report [6]; across the six countries modelled here, the number of FTEs required per 10,000 women of reproductive age is between 0 and 20% higher than that estimated in SoWMy 2014. This is because the analysis in this paper is based on allocation according to competencies, and a more extensive list of SRMNAH interventions, including those identified in the 2014 Lancet Series on Midwifery [7] as well as the PMNCH list of essential RMNCH interventions used in SoWMy 2014 [12].

For these six countries, we also compared our results to the number of midwives required per 175 births according to the 2005 World Health Report threshold. This analysis estimates a need for between 1.7 and 1.9 midwives (or nurse-midwives) per 175 births to provide essential antenatal, delivery and postnatal interventions, i.e. more than 1 per 175 births. This number is fairly constant across the six countries, mainly because it is not influenced by differences in fertility rates. It is important to note that, contrary to the rest of the paper, this estimate does not take into account the SRMNAH services needed by women and adolescents who are not pregnant (e.g. family planning, HIV prevention) to make it comparable with the established benchmark. Once these activities are taken into account, the number of midwives needed is much higher.

To put these results into a broader context, we can also consider them alongside recent analysis by WHO, estimating that countries need a minimum of 44.5 skilled health professionals per 10,000 population [2]. The middle column of Table 5 shows how this threshold translates to numbers of health professionals needed in each of our 6 countries in 2012. The fourth column shows how many SRMNAH FTEs were required in 2012 according to our ‘Dream Team’ method. This analysis suggests that the SRMNAH workforce should account for between 13 and 21% of the total health professional workforce, depending on the country’s demography and epidemiology.

**Table 5:** Need for SRMNAH workers in the context of overall need for health workers

Country	2012 population (millions)	No of health professionals needed	No of SRMNAH FTEs needed	% of needed health workforce that are FTE SRMNAH workers
Azerbaijan	9.3	41 425	5 496	13
Malawi	16.8	74 890	15 715	21
Myanmar	53.7	239 049	37 329	16
Peru	30.8	136 922	21 391	16
Uzbekistan	29.3	130 496	18 154	14
Zambia	15.0	66 843	14 295	21

**Fig. 2:**
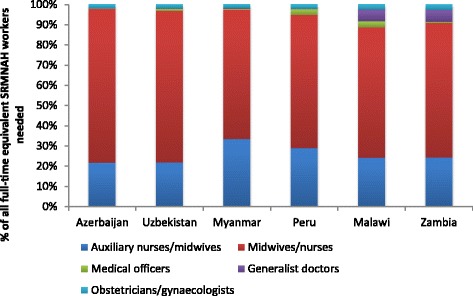
Proportion of need for full-time equivalent SRMNAH workers that can be met by different cadres, 2012

Figure 2 shows some variation in the composition of the Dream Team across countries. Between two thirds and three quarters of the FTEs required are midwives/nurse-midwives, and between a quarter and a third are auxiliary midwives/nurse-midwives. In Malawi and Zambia about 10% of the required FTEs are doctors (including obstetricians/gynaecologists), whereas in the other four countries just 2–5% of the required FTEs are doctors. In all six countries, obstetricians/gynaecologists account for about 2–3% of the required SRMNAH workforce. It should be noted that these estimates are based on the assumption that midwives/nurse-midwives and auxiliary midwives/nurse-midwives are competent to perform interventions as set out in Appendix Table 7, which will be the case if they have been educated to perform the ICM Essential Competencies in schools that adhere to global midwifery education standards [22].

The ideal composition of the SRMNAH workforce varies according to epidemiological and demographic diversity between countries. In relation to epidemiology, Malawi and Zambia need a higher share of generalist doctors because this cadre is largely responsible for management of HIV under the method of task allocation used for this analysis (see Appendix Table 7). Peru and Malawi have a higher need for medical officers due to high rates of female sterilisation as a method of contraception (see Table 4). Azerbaijan, on the other hand, needs hardly any non-specialist doctors to provide SRMNAH interventions, because of low HIV prevalence and low rates of female sterilisation. The uniform share of obstetricians and gynaecologists across all countries is due to the fact that the conditions requiring intervention from an obstetrician/gynaecologist (e.g. eclampsia) are fairly equally prevalent in all six countries (although this is partly due to limitations in the epidemiological data—see ‘Limitations’ section below).

**Fig. 3:**
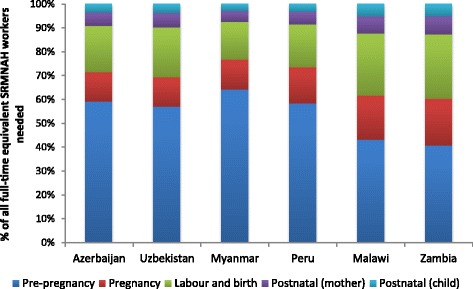
Proportion of need for full-time equivalent SRMNAH workers that occurs at each stage of the continuum of care, 2012

In relation to demography, Fig. 3 shows that the two highest-fertility countries (Malawi and Zambia) have a higher need for interventions relating to pregnancy, delivery and postnatal care. In the other four countries, more than 50% of the need occurs at the pre-pregnancy stage. These results might suggest that fertility levels have an impact on the ideal composition as well as the size of the SRMNAH workforce. However, midwives/nurse-midwives and auxiliaries (if educated according to global standards) have the competencies to deliver most of the interventions at all stages of the continuum of care. Therefore, in countries where they are needed less at the pregnancy, labour/birth and postnatal stages, they are needed more at the pre-pregnancy stage and therefore the overall proportion of need for SRMNAH workers that can be met by midwives/nurse-midwives and auxiliaries is fairly constant.

This analysis shows that, while demographic factors such as fertility have a much larger impact than epidemiological factors on the number of FTEs needed, it is epidemiological factors such as HIV prevalence and contraceptive method mix that influence the ideal composition of the SRMNAH workforce. Therefore, changes to these epidemiological and socio-cultural factors (e.g. the authorisation of a new method of contraception) are highly likely to influence the ideal composition of the health workforce.

**Fig. 4:**
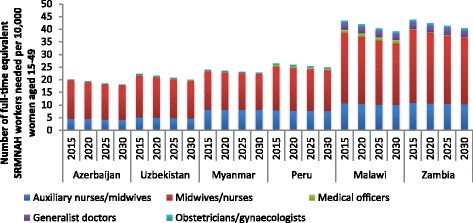
Number of full-time equivalent SRMNAH workers needed per 10,000 women of reproductive age, 2015 to 2030

Relative to the projected numbers of women of reproductive age, it is estimated that all six countries will need fewer FTEs per 10,000 women of reproductive age in 2030 than in 2015, especially Malawi and Zambia (see Fig. 4). This is largely due to an expected decrease in fertility over this period. Further, it is expected that there will be a change in the share of the workload devoted to different stages of the continuum of care, with more need for FTEs to provide pre-pregnancy services and less need at the other stages of the continuum. As noted above, however, because midwives/nurse-midwives provide services along the entire continuum of care, it is projected that the proportion of the FTEs needed per 10,000 women of reproductive age accounted for by the different cadres will not change much between 2015 and 2030.

**Fig. 5:**
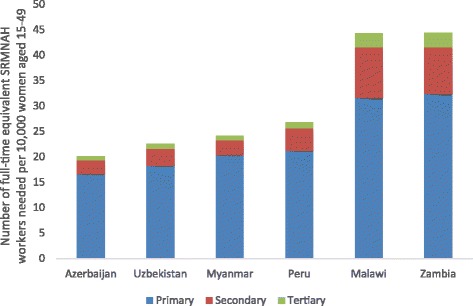
Number of full-time equivalent SRMNAH workers needed per 10,000 women of reproductive age, by level of health system, 2012

The potential of the midwifery and nursing cadres to meet most of the need for SRMNAH services is emphasised in Fig. 5, which shows that, in Azerbaijan, Uzbekistan, Myanmar and Peru, about 80% of the need for FTEs is at the primary level of care (on the assumption that, if an intervention can be delivered at all three levels of care (e.g. if mild cases are treated at primary level and more severe cases are referred to higher levels of care), 60% of the time requirement would be at the primary level of care, 30% at secondary level and 10% at tertiary level). In Malawi and Zambia this proportion is slightly lower (71 and 73% respectively), which is due to relatively high incidence of conditions such as HIV and malaria, which often require referral to higher levels of care.

### Limitations

This paper should be considered a proof of concept rather than an accurate estimate of the current situation in the six countries, given that most of the data are from 2012. The analysis does not consider the various models of service delivery (e.g. facility-based versus community-based care, obstetrician-led care versus midwife-led care) but could be further developed to accommodate these models, as well as other national or subnational variations. The future projections presented here assume no change in epidemiological conditions such as HIV prevalence. This is unlikely, which means that future national estimates will need to be based on the most recent available data and possibly modelled estimates of future disease prevalence.

Also, limitations in the epidemiological data can impact the kinds and number of interventions needed from given SRMNAH cadres. For example, the relatively similar need for interventions from obstetrician/gynaecologists across the six countries can be the result of similar prevalence rates in those countries, rather than reality on the ground in those countries.

One of the main advantages of using FTEs over headcounts in workforce modelling is that FTEs adequately convert headcounts to actual working time by taking into account each employee’s full-time/part-time working status. However, FTEs also have limitations. For example, FTEs assume that the number of employees and the number of hours worked by these employees are perfect substitutes, which is often not a realistic assumption. In addition, FTEs overlook the possible impact on workforce productivity of switching between full-time and part-time employees. This impact has been documented in the literature [23]. Of note is that this is also a limitation of headcounts.

A next step in the validation of this method would be to test it using data from high-income countries, and with different epidemiological assumptions, e.g. HIV prevalence or Caesarean section rates, to further develop the precision with which it can estimate current and future staffing needs.

One important additional component would be to estimate the costs involved in different staffing models. Being able to try out various costed staff configurations would allow countries to identify the one that gives the best return on investment.

When undertaking the analysis of time investment, it became clear that providing many of the QMNC-effective practices does not necessarily require more time (see Appendix Table 6), but rather a different way of providing care that supports the women and their newborns through the continuum of care, is tailored to the situation and needs of women and focused on strengthening their capabilities to take care of themselves and their families. This investment in women’s health and wellbeing contributes positively to strengthening communities, increasing girl’s access to education and women’s economic empowerment [24]. The way in which this approach to care can be implemented was not directly modelled but further work should highlight time savings in other areas of the continuum.

## Discussion

Traditionally, health workforce planning is based on headcounts rather than the needs of the specific population being served. SoWMy 2014 improved on headcounts by considering the amount of time that staff actually spend on providing care and allocating staff to interventions by prioritising the least expensive competent cadres. Rather than simply matching the available workforce to existing need, this paper takes a more sophisticated approach by considering the most up to date intervention package for SRMNAH, considering levels of care delivery, matching staff competence and then allocating tasks to the lowest-paid staff members competent to perform them. It thus estimates the ideal composition (the Dream Team) and complements existing planning tools such as OneHealth [15] which are effective at helping to plan deployment of the existing workforce. Additionally, the model’s basis on the latest evidence on effective SRMNAH interventions will help countries to avoid over-medicalisation of pregnancy and childbirth, which has been shown to contribute to harm [7].

Midwives/nurse-midwives who are educated according to established global standards can meet the majority of the need for SRMNAH workers. This holds true across all six of the countries featured here, despite their varying demographic and epidemiological profiles. In addition, obstetricians/gynaecologists and generalist doctors are needed to deliver some of the more specialised SRMNAH interventions, mainly because this requires a higher-level facility. This team of health care providers will be most effective if it operates within a fully enabled health system/work environment (which is far from true in many countries) and each member works to their full scope of practice with as little as possible overlap with other team members. Consideration of the optimal allocation of SRMNAH staff according to competency should lead to more efficient resource allocation and ultimately better health outcomes even in countries with less well developed health systems.

With universal health care as the main SDG for health over the next 15 years, this method can help countries further fine-tune the number and deployment of doctors, midwives and nurses. This additional level of precision will not only support getting the right services by the right staff to the right people at the right time, but will also inform the education, regulation and recruitment/deployment methods required to establish and maintain an efficient SRMNAH workforce. Obviously, the method can also be applied to health services other than SRMNAH.

In the context of the renewed focus on Universal Health Coverage (UHC), underpinned by the new annual rates of mortality reduction agreed in Ending Preventable Maternal Mortality [25] and the Every Newborn Action Plan [26], there is much to do. In many countries this calls for significant leaps in ambition and effectiveness that can feel paralysing, given the available financial and human resources. Using this new method may support the development of effective workforce models that combine quality care with solid return on investment, because tasks are allocated to the least expensive cadres competent to perform them, rather than to whichever cadres are available at whatever cost.

At the international level, WHO estimates that a needs-based shortage of health workers to achieve and sustain the SDGs could total 18 million by 2030, in mostly low- and middle-income countries [2]. The Dream Team method can provide country-specific efficient workload projections that contribute to the development and implementation of transformative national and global strategies towards achievement of the Global Strategy on Human Resources for Health: Workforce 2030 [2] (adopted at the 69th World Health Assembly in May 2016). It can also generate country-specific indicators to add to the first report of the UN High-Level Commission on Health Employment and Economic Growth [27] that proposed new actions on health employment towards achieving UHC and broader socio-economic gains.

## Conclusions

Providing high-quality care, including family planning, can save lives of women and newborns [28]. Efficient and cost-effective workforce planning means that this care can be delivered to those who need it despite finite resources. SoWMy 2014 showed that it is possible to move beyond static benchmarks and thresholds for workforce planning by taking into account the effect of demography and epidemiology on the level of need for different health care professionals. Adding allocation of interventions to specific cadres of health care professional according to their competencies represents an efficient way to allocate resources and improve quality of care.

## Appendix

**Table 6:** Essential interventions and effective practices included in the modelling, and estimated time requirement for delivery of each

Intervention	Time requirement per woman/girl/newborn (minutes)	Level(s) of care at which intervention delivered		
		Primary	Secondary	Tertiary
Pre-pregnancy				
Family planning advice^a^	20	✓	✓	
Delivery of condoms, vaginal barrier, vaginal tablet, other	15	✓		
Delivery of contraceptive pills, injectables	20	✓		
Delivery of contraceptive implants	40	✓		
Delivery of intrauterine devices (IUDs)	35	✓		
Female and male sterilisation	70		✓	
Prevention of HIV in all women of reproductive age^a^	45	✓		
Prevention of other sexually transmitted infections (STIs) in all women of reproductive age^a^	14	✓		
Syphilis management^a^	15	✓	✓	✓
Gonorrhoea management^a^	15	✓	✓	✓
Chlamydia management^a^	15	✓	✓	✓
Trichomoniasis management^a^	15	✓	✓	✓
HIV management^a^	240	✓	✓	✓
Folic acid fortification/supplementation	10	✓		
Pregnancy				
Iron and folic acid supplementation, zinc supplementation, advice to increase dietary energy and protein intake/microsupplementation/interventions to increase frequency and ease of defecation	15	✓		
Tetanus toxoid vaccination	5	✓		
Prevention of malaria with insecticide treated nets (ITNs) and antimalarial medication	5	✓		
Management of malaria with ITNs and antimalarial medication	4	✓	✓	✓
Screening for HIV for prevention of mother-to-child transmission	30	✓	✓	✓
Treatment of HIV for prevention of mother-to-child transmission	280	✓	✓	✓
Prevention of STIs as part of antenatal care	6	✓		
Gonorrhoea management^a^	10	✓	✓	✓
Chlamydia management^a^	10	✓	✓	✓
Trichomoniasis management^a^	10	✓	✓	✓
Screening for syphilis^a^	15	✓	✓	✓
Treatment of syphilis	10	✓	✓	✓
Antibiotics for treating bacterial vaginosis	10	✓	✓	✓
Antibiotics for treating asymptomatic bacteriuria	10	✓	✓	✓
Treatment for symptomatic urinary tract infections	10	✓	✓	✓
Any topical treatment for vaginal candidiasis	10	✓	✓	✓
Calcium supplementation to prevent hypertension	8	✓	✓	✓
Interventions for cessation of smoking^a^	16	✓		
Antihypertensive drugs to treat high blood pressure (including low-dose aspirin to prevent pre-eclampsia)^a^	50	✓	✓	✓
Magnesium sulphate for eclampsia (nurse or midwife)	180	✓	✓	✓
Magnesium sulphate for eclampsia (ob/gyn)	60		✓	✓
Antibiotics for prevention of preterm premature rupture of membranes	30	✓	✓	✓
Corticosteroids to prevent respiratory distress (nurse or midwife)	40	✓	✓	✓
Corticosteroids to prevent respiratory distress (generalist doctor)	30	✓	✓	✓
Safe abortion (vacuum aspiration or dilation & curettage)	30		✓	✓
Post-abortion care (auxiliary)	90	✓	✓	
Post-abortion care (nurse or midwife)	90	✓	✓	✓
Post-abortion care (ob/gyn)	30	✓	✓	✓
Reduce malpresentation at term with external cephalic version^a^	107		✓	✓
Induction of labour to manage pre-labour rupture of membranes at term	86		✓	✓
Digital perineal massage	0	✓	✓	✓
Interventions intended to promote breastfeeding	30	✓	✓	✓
Anti-D administration in pregnancy for preventing rhesus alloimmunisation	1	✓		
Antiplatelet agents for preventing pre-eclampsia and its complications	30	✓		
Interventions for preventing and treating pelvic and back pain in pregnancy	15	✓		
Psychological and psychosocial interventions for preventing postpartum depression	30	✓		
Labour and birth				
Normal labour and delivery management and social support during childbirth (nurse or midwife)	360	✓	✓	✓
Normal labour and delivery management and social support during childbirth (ob/gyn)	30	✓	✓	✓
Any perineal technique during the second stage of labour	0	✓	✓	✓
Immersion in any bathtub or pool during labour	0	✓	✓	✓
Upright positions assumed by women in first stage of labour	0	✓	✓	✓
Acupuncture or acupressure for pain management in labour	0	✓	✓	✓
Massage, reflexology and other manual methods for pain management in labour	0	✓	✓	✓
Relaxation techniques	0	✓	✓	✓
Restrictive episiotomy	0	✓	✓	✓
Any inhaled analgesia during labour	0	✓	✓	✓
Active management of third stage labour (to deliver placenta) to prevent postpartum haemorrhage (including uterine massage, uterotonics and controlled cord traction)	10	✓	✓	✓
Oxytocin given prophylactically for third stage of labour	0	✓	✓	✓
Prophylactic use of ergot alkaloids in third stage of labour	0	✓	✓	✓
Rapid versus stepwise negative pressure application for vacuum extraction	0	✓	✓	✓
Screen for HIV during childbirth if not already tested^a^	11	✓	✓	✓
Manage HIV during childbirth if not already tested^a^	120	✓	✓	✓
Caesarean section for maternal/foetal indication (including prophylactic antibiotic for C-section) - auxiliary^a^	210		✓	✓
Caesarean section for maternal/foetal indication (including prophylactic antibiotic for C-section) - nurse or midwife^a^	90		✓	✓
Caesarean section for maternal/foetal indication (including prophylactic antibiotic for C-section) – ob/gyn^a^	90		✓	✓
Induction of labour for prolonged pregnancy (midwife or nurse)	40		✓	✓
Induction of labour for prolonged pregnancy (ob/gyn)	20		✓	✓
Management of postpartum haemorrhage (PPH) (manual removal of placenta and/or surgical procedures and/or oxytocics)-auxiliary	120		✓	✓
Management of PPH (manual removal of placenta and/or surgical procedures and/or oxytocics)-nurse or midwife	60		✓	✓
Management of PPH (manual removal of placenta and/or surgical procedures and/or oxytocics)-ob/gyn	90		✓	✓
Interventions intended to promote breastfeeding	30	✓	✓	✓
Alternative institutional birth environment	0	✓	✓	✓
Midwife-led continuity models of care	0	✓	✓	✓
Labour assessment programmes aimed at delaying admission to the labour ward	0	✓	✓	✓
Postnatal: mother				
Postnatal preventive care	80	✓	✓	✓
Detect and treat postpartum sepsis (auxiliary)	150	✓	✓	✓
Detect and treat postpartum sepsis (nurse or midwife)	60	✓	✓	✓
Continuous versus interrupted sutures for episiotomy/ second degree tears	0	✓	✓	✓
Psychological and psychosocial interventions for preventing postpartum depression	30	✓	✓	✓
Single administration of paracetamol for early postpartum pain	5	✓	✓	✓
Any type of approved analgesia for after-birth pains (vaginal birth)	0	✓	✓	✓
Antibiotic regimens for endometritis after delivery	15	✓	✓	✓
Analgesic rectal suppositories for relief of perineal pain	0	✓	✓	✓
Postnatal: newborn				
Neonatal resuscitation with bag and mask^a^	20	✓	✓	✓
Kangaroo Mother Care	30	✓	✓	✓
Skin-to-skin contact between mother and baby	5	✓	✓	✓
Immediate thermal care	5	✓	✓	✓
Extra support for feeding small and preterm babies^a^	90	✓	✓	✓
Management of newborns with jaundice	150	✓	✓	✓
Initiate prophylactic antiretroviral therapy (ART) for babies exposed to HIV^a^	30	✓	✓	✓
Presumptive antibiotic therapy for newborns at risk of bacterial infections^a^	40	✓	✓	✓
Surfactant to prevent respiratory distress syndrome in preterm babies^a^	60	✓	✓	✓
Continuous positive airway pressure (CPAP) to manage babies with respiratory distress syndrome^a^	120	✓	✓	✓
Interventions intended to promote breastfeeding	60	✓	✓	✓
Exclusive breastfeeding for at least 6 months	0	✓	✓	✓

^a^Not included in OneHealth so the time estimate was an expert opinion

**Table 7:** Allocation of tasks to cadres

Intervention	Auxiliary midwife/nurse-midwife	Midwife/ Nurse-midwife	Medical officer	Doctor (generalist)	Obstetrician/Gynaecologist
Pre-pregnancy					
Family planning advice	✓				
Delivery of condoms, vaginal barrier, vaginal tablet, other	✓				
Delivery of contraceptive pills, injectables	✓				
Delivery of contraceptive implants		✓			
Delivery of intrauterine devices (IUDs)		✓			
Female and male sterilisation			✓		
Prevention of HIV in all women of reproductive age		✓			
Prevention of other sexually transmitted infections (STIs) in all women of reproductive age		✓			
Syphilis management		✓			
Gonorrhoea management		✓			
Chlamydia management		✓			
Trichomoniasis management		✓			
HIV management				✓	
Folic acid fortification/supplementation		✓			
Pregnancy					
Iron and folic acid supplementation, zinc supplementation, advice to increase dietary energy and protein intake/microsupplementation/interventions to increase frequency and ease of defecation		✓			
Tetanus toxoid vaccination		✓			
Prevention of malaria with insecticide treated nets (ITNs) and antimalarial medication		✓			
Management of malaria with ITNs and antimalarial medication		✓			
Screening for HIV for prevention of mother-to-child transmission		✓			
Treatment of HIV for prevention of mother-to-child transmission		✓			
Prevention of STIs as part of antenatal care		✓			
Gonorrhoea management		✓			
Chlamydia management		✓			
Trichomoniasis management		✓			
Screening for syphilis		✓			
Treatment of syphilis		✓			
Antibiotics for treating bacterial vaginosis		✓			
Antibiotics for treating asymptomatic bacteriuria		✓			
Treatment for symptomatic urinary tract infections		✓			
Any topical treatment for vaginal candidiasis		✓			
Calcium supplementation to prevent hypertension		✓			
Interventions for cessation of smoking	✓				
Antihypertensive drugs to treat high blood pressure (including low-dose aspirin to prevent pre-eclampsia)		✓			
Magnesium sulphate for eclampsia (nurse or midwife)		✓			
Magnesium sulphate for eclampsia (ob/gyn)					✓
Antibiotics for prevention of preterm premature rupture of membranes		✓			
Corticosteroids to prevent respiratory distress (nurse or midwife)		✓			
Corticosteroids to prevent respiratory distress (generalist doctor)				✓	
Safe abortion (vacuum aspiration or dilation & curettage)		✓			
Post-abortion care (auxiliary)	✓				
Post-abortion care (nurse or midwife)		✓			
Post-abortion care (ob/gyn)					✓
Reduce malpresentation at term with external cephalic version*		✓			
Induction of labour to manage pre-labour rupture of membranes at term		✓			
Digital perineal massage		✓			
Interventions intended to promote breastfeeding	✓				
Anti-D administration in pregnancy for preventing rhesus alloimmunisation		✓			
Antiplatelet agents for preventing pre-eclampsia and its complications		✓			
Interventions for preventing and treating pelvic and back pain in pregnancy		✓			
Psychological and psychosocial interventions for preventing postpartum depression		✓			
Labour and birth					
Normal labour and delivery management and social support during childbirth (nurse or midwife)		✓			
Normal labour and delivery management and social support during childbirth (ob/gyn)					✓
Any perineal technique during the second stage of labour		✓			
Immersion in any bathtub or pool during labour		✓			
Upright positions assumed by women in first stage of labour		✓			
Acupuncture or acupressure for pain management in labour		✓			
Massage, reflexology and other manual methods for pain management in labour		✓			
Relaxation techniques		✓			
Restrictive episiotomy		✓			
Any inhaled analgesia during labour		✓			
Active management of third stage labour (to deliver placenta) to prevent postpartum haemorrhage (including uterine massage, uterotonics and controlled cord traction)		✓			
Oxytocin given prophylactically for third stage of labour		✓			
Prophylactic use of ergot alkaloids in third stage of labour		✓			
Rapid versus stepwise negative pressure application for vacuum extraction		✓			
Screen for HIV during childbirth if not already tested	✓				
Manage HIV during childbirth if not already tested		✓			
Caesarean section for maternal/foetal indication (including prophylactic antibiotic for C-section) - auxiliary	✓				
Caesarean section for maternal/foetal indication (including prophylactic antibiotic for C-section) - nurse or midwife		✓			
Caesarean section for maternal/foetal indication (including prophylactic antibiotic for C-section) – ob/gyn					✓
Induction of labour for prolonged pregnancy (midwife or nurse)		✓			
Induction of labour for prolonged pregnancy (ob/gyn)					✓
Management of postpartum haemorrhage (PPH) (manual removal of placenta and/or surgical procedures and/or oxytocics)-auxiliary	✓				
Management of PPH (manual removal of placenta and/or surgical procedures and/or oxytocics)-nurse or midwife		✓			
Management of PPH (manual removal of placenta and/or surgical procedures and/or oxytocics)-ob/gyn					✓
Interventions intended to promote breastfeeding	✓				
Alternative institutional birth environment		✓			
Midwife-led continuity models of care		✓			
Labour assessment programmes aimed at delaying admission to the labour ward		✓			
Postnatal: mother					
Postnatal preventive care		✓			
Detect and treat postpartum sepsis (auxiliary)	✓				
Detect and treat postpartum sepsis (nurse or midwife)		✓			
Continuous versus interrupted sutures for episiotomy/ second degree tears		✓			
Psychological and psychosocial interventions for preventing postpartum depression		✓			
Single administration of paracetamol for early postpartum pain		✓			
Any type of approved analgesia for after-birth pains (vaginal birth)		✓			
Antibiotic regimens for endometritis after delivery		✓			
Analgesic rectal suppositories for relief of perineal pain		✓			
Postnatal: newborn					
Neonatal resuscitation with bag and mask		✓			
Kangaroo mother care		✓			
Skin-to-skin contact between mother and baby	✓				
Immediate thermal care		✓			
Extra support for feeding small and preterm babies		✓			
Management of newborns with jaundice	✓				
Initiate prophylactic antiretroviral therapy (ART) for babies exposed to HIV	✓				
Presumptive antibiotic therapy for newborns at risk of bacterial infections		✓			
Surfactant to prevent respiratory distress syndrome in preterm babies		✓			
Continuous positive airway pressure (CPAP) to manage babies with respiratory distress syndrome		✓			
Interventions intended to promote breastfeeding	✓				
Exclusive breastfeeding for at least 6 months	✓

**Table 8:** Sources of data on incidence of conditions requiring interventions listed among the Lancet Series on Midwifery effective practices

Bacterial vaginosis	Based on Mullick et al 2005 (http://www.ncbi.nlm.nih.gov/pmc/articles/PMC1745010/pdf/v081p00294.pdf), prevalence is variable, but up to 50% in Sub-Saharan Africa: Uganda (*n* = 4,033) prevalance = 48.5% Zimbabwe (*n* = 1,656) prevalence = 4.3% Central African Republic (*n* = 481) prevalance = 21.1% Kenya (*n* = 621) prevalence = 9% Tanzania (*n* = 660) prevalance = 24% Kenyon et al (2013) reported data for a number of countries: http://www.sciencedirect.com/science/article/pii/S000293781300478X If country-specific data were found in either of the above sources, those data were used. Otherwise, 29% was assumed, based on a US study: http://journals.lww.com/greenjournal/Abstract/2007/01000/Prevalence_of_Bacterial_Vaginosis__2001_2004.18.aspx
Asymptomatic bacteruria	Allsworth et al (2007) reported incidence of between 2 and 10% (in developing countries): http://citeseerx.ist.psu.edu/viewdoc/download?doi=10.1.1.463.1909&rep=rep1&type=pdf 6% was assumed for all 6 countries [(2 + 10/2)]
Symptomatic UTI	Nabbugodi et al (2015) reported incidence of between 12 and 40% overall: http://www.agialpress.com/journals/oajost/2015/101115/ 26% was assumed for all 6 countries [(12 + 40)/2]
Vaginal candidiasis	Marai (2001) reported incidence of between 14 and 42%: http://www.ajol.info/index.php/eamj/article/viewFile/8947/1553 Ibrahim et al (2013) reported incidence of 41% in Nigeria: http://www.ncbi.nlm.nih.gov/pubmed/23829126 28% was assumed for all 6 countries [(14 + 42)/2]
First time births	Expert opinion: 50% in middle income countries and 40% low-income countries
Pelvic and back pain	Pierce et al (2012) estimated 71% experience lumbopelvic pain during pregnancy in Australia

## Abbreviations

DHS: Demographic and health survey
ECV: External cephalic version
FTE: Full-time equivalent
HDI: Human development index
HRH: Human resources for health
MICS: Multiple indicator cluster survey
MMR: Maternal mortality ratio
MNH: Maternal and child health
MoH: Ministry of Health
PMNCH: Partnership for Maternal, Newborn and Child Health
QMNC: Quality maternal and newborn care
RMNCH: Reproductive, maternal, newborn and child health
SDG: Sustainable development goal
SoWMy: State of the World’s Midwifery
SRMNAH: Sexual, reproductive, maternal, newborn and adolescent health
UHC: Universal health coverage
UN: United Nations
UNAIDS: Joint United Nations Programme on HIV/AIDS
WHO: World Health Organization

## Footnotes

1 The health care professional cadres included in the Dream Team are Auxiliary midwives and auxiliary nurse midwives/ Midwives and nurse-midwives/medical officers/generalists/obstetricians and gynaecologists, further information is provided in Table 7 in the Appendix.
